# P-1540. Virulence Analysis of Type VI Secretion System in Neonatal Meningitis E. coli using Galleria mellonella as an Infection Model

**DOI:** 10.1093/ofid/ofaf695.1721

**Published:** 2026-01-11

**Authors:** Julia Ienes-Lima, Ariana Martinez, Aline de Oliveira, Lisa Nolan, Catherine Logue

**Affiliations:** University of Georgia, Athens, GA; University of Georgia, Athens, GA; University of Georgia, Athens, GA; University of Georgia, Athens, GA; University of Georgia, Athens, GA

## Abstract

**Background:**

Neonatal meningitis *E. coli* (NMEC) is a common cause of bacterial meningitis in newborns worldwide. NMEC replicates within macrophages, survives in the bloodstream, and traverses the blood-brain barrier, resulting in meningitis. Most NMEC cases are associated with serogroups O18, O83, O7, O12, O1, and O45. Two clusters of the Type 6 secretion system (T6SS) (T6SS-1 and T6SS-2) were identified in NMEC O18:K1. Studies demonstrated that the T6SS plays a key role in pathogenesis and biofilm formation of NMEC, though the function of specific T6SS genes remains unclear. This study aimed to investigate the T6SS role in NMEC virulence through *in vivo* infection of *Galleria mellonella* larvae, hypothesizing that deletion of T6SS genes would reduce strain virulence.

**Figure 1.**
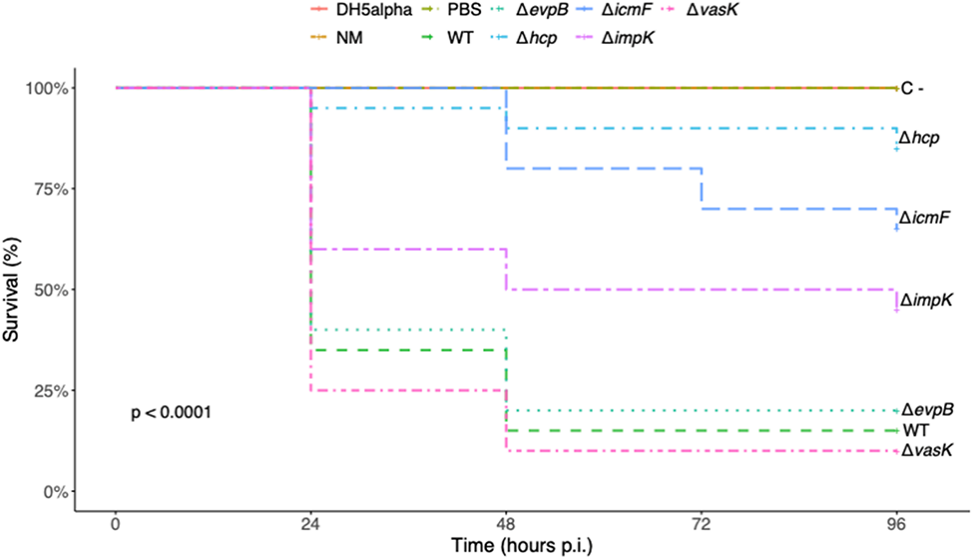
Kaplan-Meier survival curve of G. mellonella larvae infected with T6SS mutant strains.

**Figure 2.**
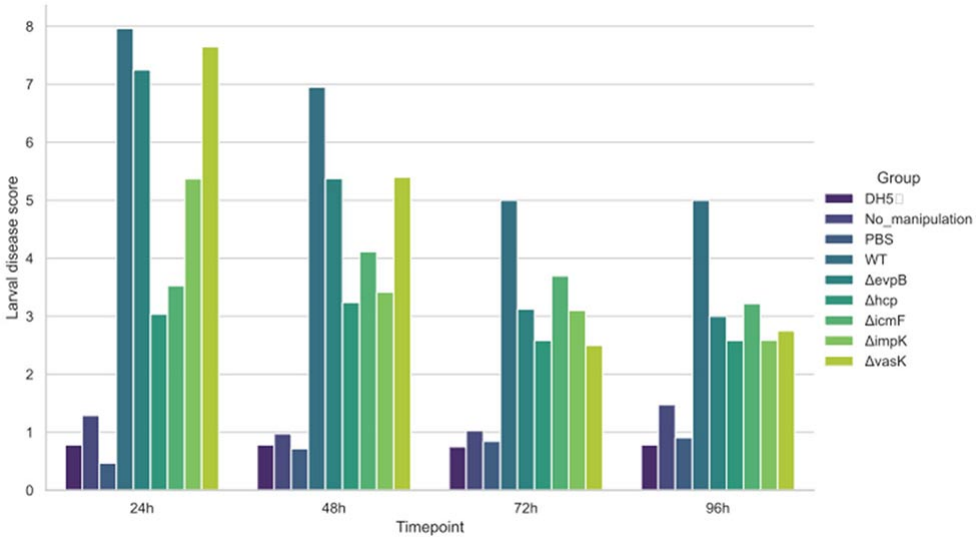
Larval Disease Score (LDS) of larvae infected with T6SS mutant strains throughout the 96h observation period.

**Methods:**

An NMEC O18 strain was used to construct T6SS mutants lacking the *hcp, icmF, impK, evpB,* and *vasK* genes. Strains belonging to eight different serogroups and distinct T6SS operon structures (O1, O12, O16, O18, O78, O83, O7 and O45) were also analysed. Larvae were infected with 10^6^ CFU/mL of each bacterial strain suspension and incubated at 37 °C for 96 hours. PBS was used as a negative control, along with a non-infected group, and a non-pathogenic *E. coli* strain (DH5α). Larval activity, melanization, and cocoon production were evaluated and used to calculate the larval disease score (LDS).

**Figure 3.**
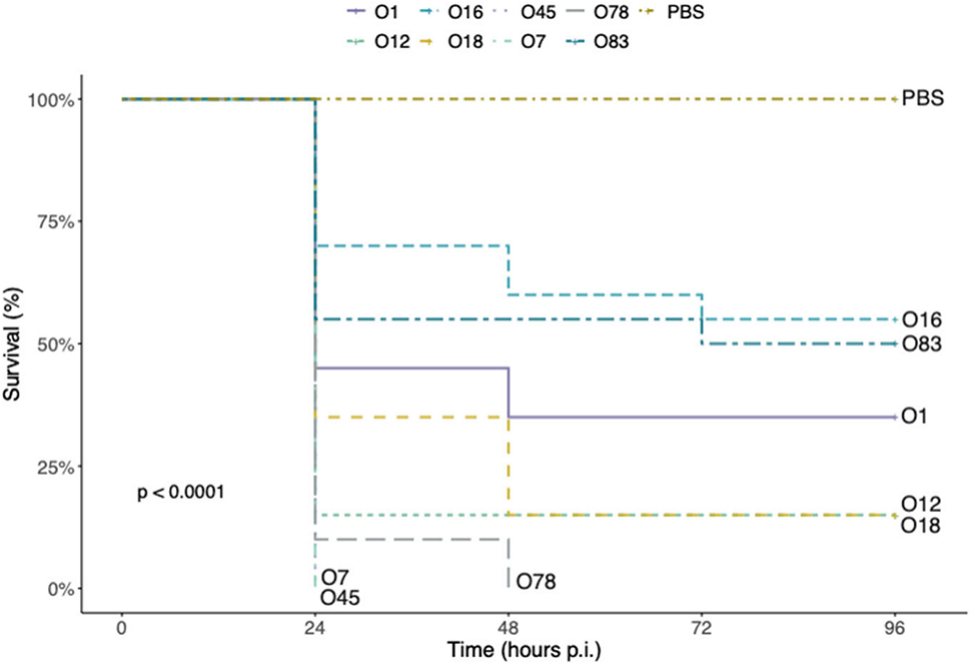
Kaplan-Meier survival curve of G. mellonella larvae infected with NMEC strains of different serogroups.

**Figure 4.**
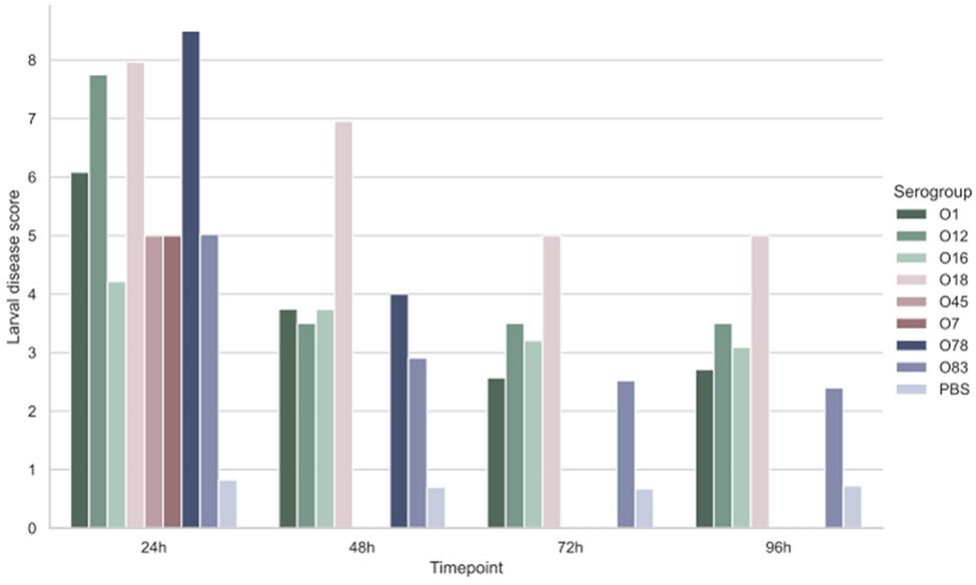
Larval Disease Score (LDS) of larvae infected with NMEC strains of different serogroups throughout the 96h observation period.

**Results:**

Deletion of *hcp* and *icmF* genes attenuated the virulence of the O18 strain, promoting larval survival rates of 85% and 65%, respectively. In contrast, only 15% of larvae infected with the wild-type strain survived. These two mutants also presented lower LDS compared to the WT. However, no attenuation was observed in larvae infected with Δ*evp* or Δ*vasK* strains. In terms of serogroup virulence, the O7 and O45 strains surprisingly promoted 100% mortality after 24h post-infection. Among the strains T6SS^+^, infections with O78, O18, and O12 resulted in low survival but higher LDS, while infections with O16 and O83 strains showed higher survival rates (55% and 50%, respectively) and lower LDS.

**Conclusion:**

These findings suggest that T6SS contributes significantly to the virulence of NMEC strains belonging to the O18 serogroup but does not play a major role in the virulence of O7 and O45 strains, which may harbor additional virulence factors.

**Disclosures:**

All Authors: No reported disclosures

